# Multisensory Rehabilitation Training Improves Spatial Perception in Totally but Not Partially Visually Deprived Children

**DOI:** 10.3389/fnint.2017.00029

**Published:** 2017-10-19

**Authors:** Giulia Cappagli, Sara Finocchietti, Gabriel Baud-Bovy, Elena Cocchi, Monica Gori

**Affiliations:** ^1^Unit for Visually Impaired People (U-VIP), Fondazione Istituto Italiano di Technologia, Genoa, Italy; ^2^Istituto Chiossone, Genova, Italy

**Keywords:** auditory perception, blindness, child development, spatial hearing, visual impairment

## Abstract

Since it has been shown that spatial development can be delayed in blind children, focused sensorimotor trainings that associate auditory and motor information might be used to prevent the risk of spatial-related developmental delays or impairments from an early age. With this aim, we proposed a new technological device based on the implicit link between action and perception: ABBI (Audio Bracelet for Blind Interaction) is an audio bracelet that produces a sound when a movement occurs by allowing the substitution of the visuo-motor association with a new audio-motor association. In this study, we assessed the effects of an extensive but entertaining sensorimotor training with ABBI on the development of spatial hearing in a group of seven 3–5 years old children with congenital blindness (*n* = 2; light perception or no perception of light) or low vision (*n* = 5; visual acuity range 1.1–1.7 LogMAR). The training required the participants to play several spatial games individually and/or together with the psychomotor therapist 1 h per week for 3 months: the spatial games consisted of exercises meant to train their ability to associate visual and motor-related signals from their body, in order to foster the development of multisensory processes. We measured spatial performance by asking participants to indicate the position of one single fixed (static condition) or moving (dynamic condition) sound source on a vertical sensorized surface. We found that spatial performance of congenitally blind but not low vision children is improved after the training, indicating that early interventions with the use of science-driven devices based on multisensory capabilities can provide consistent advancements in therapeutic interventions, improving the quality of life of children with visual disability.

## Introduction

The ability to construct a sense of space in terms of auditory spatial representation is of fundamental importance for visually impaired children. Indeed while sighted children encode spatial information according to a visual frame of reference to orient in space and engage in social interactions (Gori et al., 2016), visually impaired children strongly rely on auditory cues to independently navigate in the environment and encode spatial and social information.

Several studies assessing spatial cognition in blind adults indicate that plasticity mechanisms can act in the blind brain to compensate for the lack of vision (Pasqualotto and Proulx, 2012). Unfortunately, only few studies investigated if compensatory mechanisms occur at an early stage in the development of visually impaired children. For instance, it has been shown that blind children can identify the position of a sound source both in the horizontal and vertical dimensions and move toward it (Ashmead et al., 1998). On the contrary, other studies indicate that sound localization abilities are impaired in infants and children with severe congenital blindness (Fraiberg, 1977; Cappagli et al., 2015, 2017; Cappagli and Gori, 2016; Vercillo et al., 2016) and even motor responses to sound can be delayed (Fraiberg et al., 1966; Adelson and Fraiberg, 1974). For example, they seem to be unable to identify the position of sonorous objects embedded in space before 12 months (Fazzi et al., 2011), while sighted children start around 5 months (Bayley, 1993). Similarly, their abilities to represent the relation of sounds in space is compromised both in the horizontal (Vercillo et al., 2016) and sagittal planes (Cappagli et al., 2015). A possible explanation for such different findings is that the lack of vision causes a delay in the development of mobility and locomotor skills (Fraiberg, 1977; Troster et al., 1994; Bigelow, 1996; Tobin et al., 1997) which in turn causes visually impaired children to accumulate much less spatial experience compared to their sighted peers (Fraiberg, 1977; Warren, 1977; Landau et al., 1984). As a consequence, blind children develop good spatial hearing abilities according to the richness of perceptual experiences in their early life. For this reason, the goal of therapeutic interventions should be to increase the opportunities to explore the surrounding environment and interact with peers, in order to learn how to use hearing to establish the sensorimotor feedback that for sighted children is necessary to promote spatial development (Bremner et al., 2008).

Since the negative effects of visual impairments on perceptual development often persist in adulthood, the creation and validation of technological devices to support the development of perceptual skills in visually impaired people would guarantee an improvement of independence and an increment of exploratory opportunities. Nonetheless the development of assistive devices has been mainly targeted to visually impaired adults, principally because they are meant to compensate well-consolidated perceptual functions (Gori et al., 2016). The main reason why assistive devices created for adults are not feasible for children is that they are based on artificial operating principles that young users find difficult to learn. For example, many substitution devices based on visual-to-auditory conversion pretend to convey information about luminosity using auditory parameters such as frequency levels, which results in an artificial procedure. Nonetheless, since cortical plasticity is maximal during the first years of life (Bremner et al., 2008; Vasilyeva and Lourenco, 2012; Gori, 2015), the use of assistive devices should be adopted as soon as possible to facilitate the development of new skills. The intuitive nature of assistive devices could be the key to make their use easier for children: in this way the computational power of the brain would be fostered and motivation would be facilitated in users. In this sense, the early integration of technological devices in classical rehabilitation programs for visually impaired children would provide an immediate benefit in terms of quality of life. To our knowledge, only a preliminary study investigated the extent to which the artificial tactile stimulation produced by the sensory substitution device motivates sighted infants to explore the environment and interact with peers (Segond et al., 2007). Although sighted infants positively responded to the tactile feedback when it was contingent to their self-movement, the device hasn't been tested on blind infants. Another neglected aspect in the creation of assistive devices for visually impaired people is the implementation of features that can foster the development of social skills other than perceptual skills. Indeed, visual impairment can have negative effects not only on sensory development, but also on social interaction skills of blind children, significantly influencing the quality of life of visually impaired individuals. To our knowledge, only one platform has been created to guide social interactions in visually impaired individuals (Krishna et al., 2008). The platform is based on a face detection process and provides an audio signal that helps users understanding when someone approaches, allowing them to initiate a conversation and guiding them in making eye contacts. Nonetheless no platforms have been developed to create opportunities to interact at a young age in a group of visually impaired children.

To sum up, there is a lack of studies assessing the effects of early therapeutic interventions with technological devices on spatial development in visually impaired children. For this reason, the present study aims at testing a novel technological device that can be used as part of a rehabilitation training specifically focused on activities fostering the development of perceptual and motor abilities in order to improve the quality of life of young children with visual impairments. The technological device used for the rehabilitation intervention is the Audio Bracelet for Blind Interaction (ABBI) that produces a sound when a movement occurs by allowing the substitution of the visuo-motor association with an audio-motor association. To evaluate the efficacy of the device, a group of seven 3–5 years old children with congenital blindness (*n* = 2; light perception or no perception of light) or low vision (*n* = 5; visual acuity range 1.1–1.7 LogMAR) has been enrolled to perform a 3-months rehabilitation training during which children played several spatial games individually and/or together with the psychomotor therapist 1 h per week.

## Materials and methods

### Task and procedure

**Figure 1:**
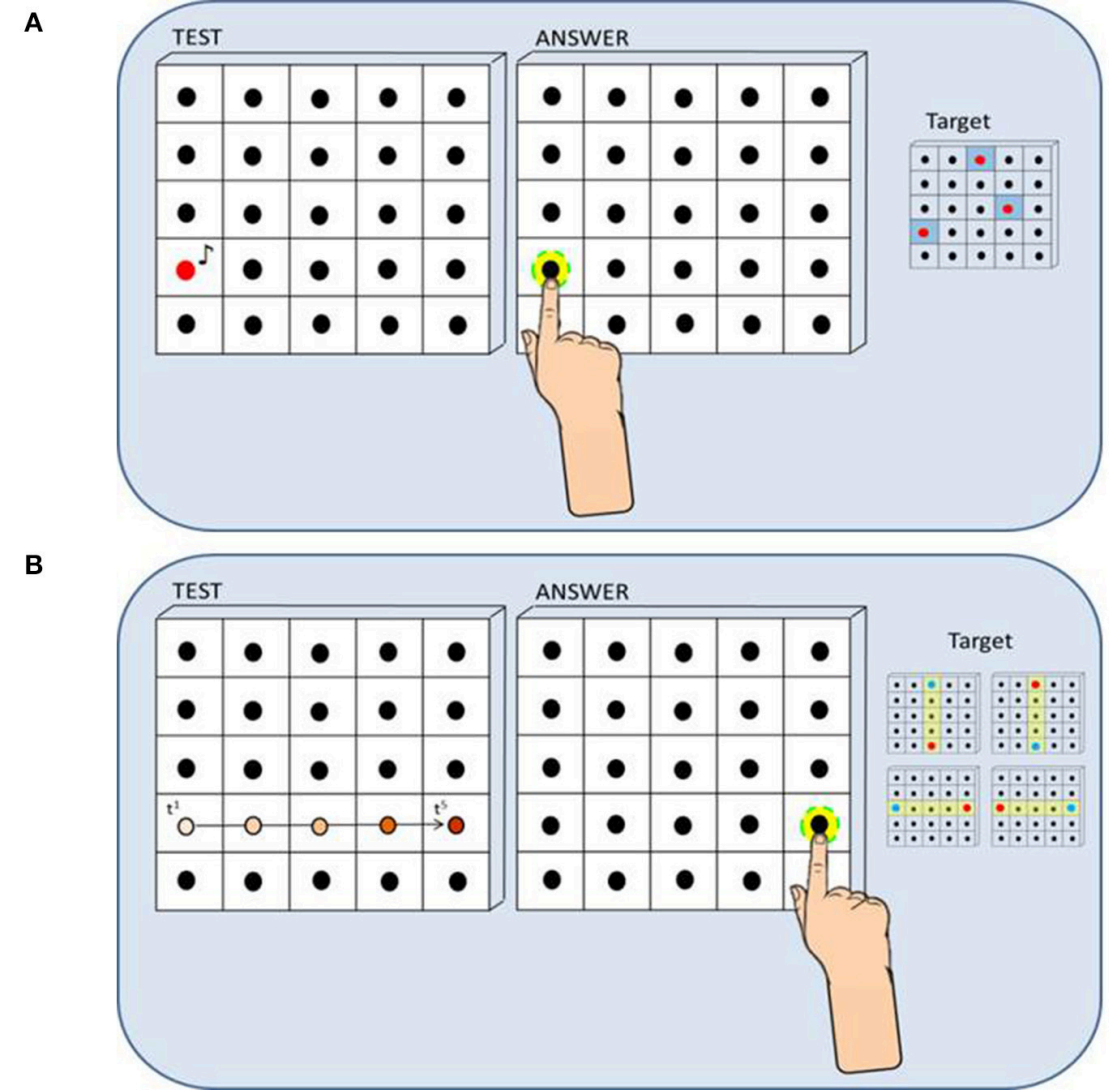
Method and procedure (the figure has been taken from Cappagli et al., 2017). **(A)** Static Sound Localization Task: In the Static Sound Localization Task, a sound coming from one out of three target loudspeakers (red dots on the right) was presented and the participant responded by touching the perceived sound source with the index finger of the dominant hand. Localization error was calculated for each trial by extracting the length of the vector that connected the actual and the perceived position of the loudspeaker (mm). **(B)** Dynamic Sound Localization Task: In the Dynamic Sound Localization Task, the sound moved across loudspeakers from a starting point (blue dots on the right) toward one of the four end-point positions (red dots on the right) and the participant responded by touching the end-point of the motion trajectory, that is the last active loudspeaker. Localization error was calculated for each trial by extracting the length of the vector that connected the actual and the perceived position of the loudspeaker (mm).

The haptic setup is made of a vertical surface (50 × 50 cm) covered by non-adjacent tactile sensors (2 × 2 cm) that can register the position of the contact and provide accurate information about spatial errors (Figure 1). The haptic setup consists of 25 blocks of sensors and 25 loudspeakers, each loudspeaker embedded in a block of sensors containing 16 tactile sensors (4 × 4). The child was seated on a chair in front of a table which supported the haptic setup used to run the experiment. Distance from the setup to the trunk was maintained at 40 cm by positioning the chair in order to make the device easily reachable with the dominant hand for all the participants. While listening to the sound presented, the participant kept the dominant hand fixed on the starting point on the table that was approximately the position corresponding to the right limit of the haptic device (the left limit for left-handed). Two auditory localization tasks have been presented. During the *static* localization task the child was asked to indicate the position of a single sound source by touching the perceived sound source position with the index finger of the dominant hand (Figure 1A). Three target loudspeakers (red dots) sufficiently distributed on the surface (left, center, right) were selected and judged equally difficult to reach in a previous pilot study: each target was sampled five times for a total of 15 trials. During the *dynamic* localization task the child was asked to indicate the direction of a sound motion in the horizontal and vertical plane by touching the end-point of a motion trajectory (Figure 1B). Four motion trajectories centered on the setup (yellow bars: up-to-down, down-to-up, left-to-right, and right-to-left) were selected: each motion trajectory was sampled four times for a total of 16 trials. On each trial, the sound moved across loudspeakers from a starting point (blue dots) toward one of the four end-point positions (red dots) and the participant indicated the last activated loudspeaker. For both tasks, the auditory stimulus was a “meow” sound registered and implemented in Matlab (R2013a, The MathWorks, USA). In the static condition of the task, a single sound was played at a time. In the dynamic condition of the task, while the first sound was playing the second sound started in order to create an audio motion. The sound has been chosen in order to make the task child-friendly: children were instructed to try and catch the kitten inside a box. For both tasks, localization error was calculated for each trial by extracting the length of the vector that connected the actual and the perceived position of the loudspeaker (mm). Spatial accuracy indicated by localization error was computed for each participant and for each group of children. For data analysis, we used the non-parametric Mann-Whitney test to compare the performance of sighted vs. low vision or early blind children, while we used the non-parametric Wilcoxon test to compare the performance in the pre-training and post-training sessions for both groups of visually impaired children. To estimate the effect size, we calculated Cohen's D Effect Size from Wilcoxon test results as d = z/sqrt(n) where n is the sample size. Tasks reliability has been investigated with the split-half method and subsequent adjustment with the Spearman–Brown prediction formula. In this case, the number of trials of each test condition in the pre-training session (*n* = 15 for the static condition, *n* = 16 for the dynamic condition) has been divided into two parts, then the two parts have been compared and correlated as if they were two separate administrations of the same task. All statistical analyses were conducted with IBM SPSS statistics Version 20 (IBM Corp. Released 2011). The index of improvement has been calculated for each group as the difference in terms of mean localization error between the pre-training and the post-training sessions and significant differences among groups have been assessed with a *t*-Student test. Before entering the experimental room, all children with normal or residual vision were blindfolded so they had no chance to see the experimental setup. Before starting the test, each child was asked to familiarize with the experimental setup by exploring it with hands for 30 s. The study was approved by the ethics committee of the local health service and parental or adult informed written consent for the study was obtained in all cases. The task and procedure are the same as in Cappagli et al. (2017) replicated before and after the training with the ABBI device.

### ABBI device: a new technological system

**Figure 2:**
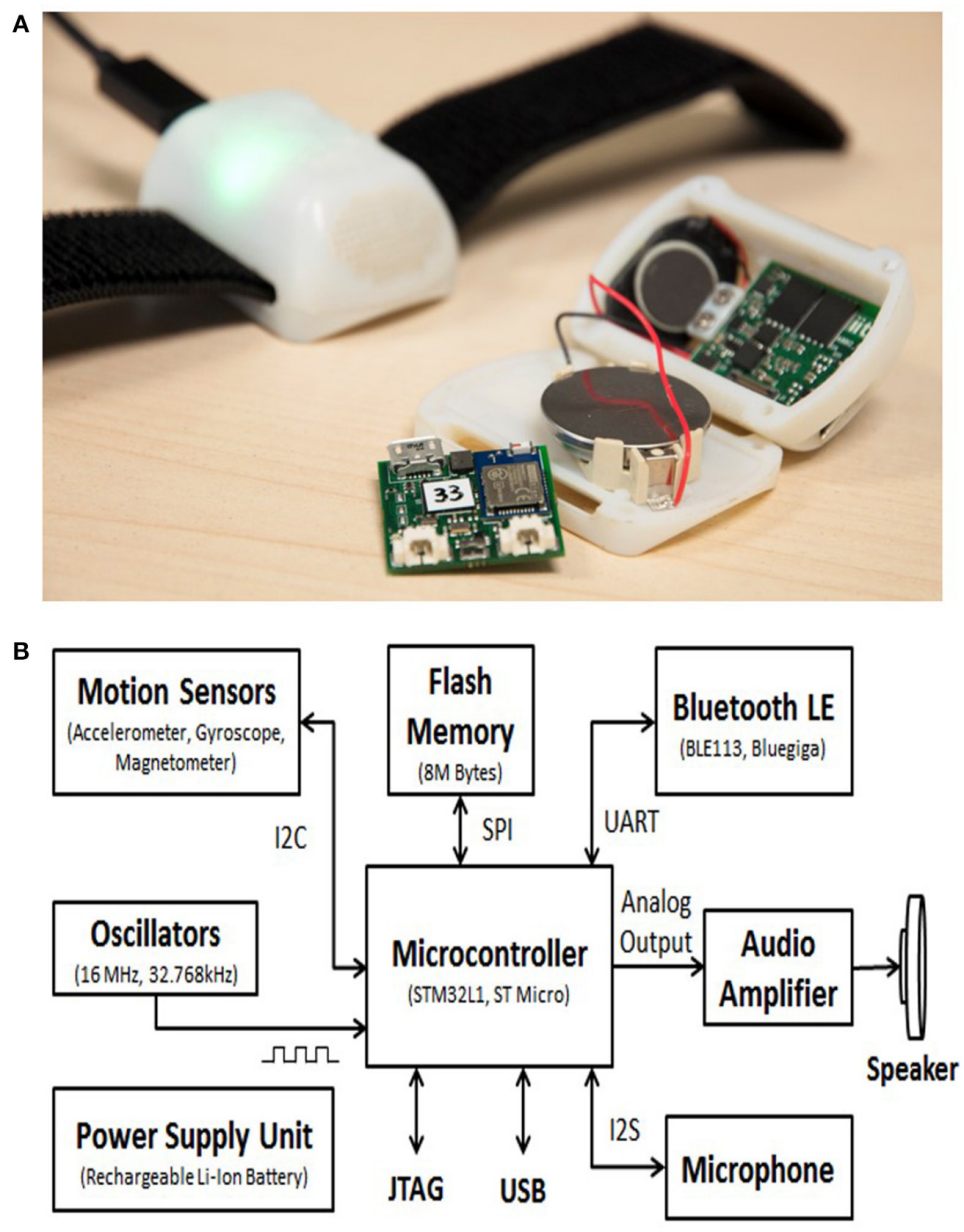
The ABBI system **(A)** ABBI bracelet. The bracelet is composed of a light and small bracelet consisting of a 3D inertial sensor, a speaker, a battery, and on-board electronics that can connect to a smartphone application wirelessly via a Bluetooth LE (Bluetooth Low Energy) link. **(B)** ABBI block diagram. The block diagram indicates the main components and their communication protocols within the ABBI bracelet.

The ABBI aims at recovering early spatial impairments in the blind child by exploiting a natural multisensory audio-motor association. The device consists of an audio bracelet connected to a smartphone that produces a sound when a movement occurs, thus it allows the substitution of the visuo-motor with the audio-motor association. In its simplest version, a simple sound is played when the acceleration exceeds an adjustable threshold marking the beginning of movement. Indeed when moving the arm, sighted children can observe and monitor their own actions through visual input. In absence of vision, the visual feedback of body movements is not available, but an auditory feedback might help representing and monitoring actions occurring in space. The ABBI device (Figure 2) is composed of a light and small bracelet that can connect to a smartphone application wirelessly via a Bluetooth LE (Blue-tooth Low Energy) link. The ABBI bracelet contains a microcontroller, an integrated audio system, motion sensors and a wireless communication module). The dimensions (55 × 35 × 25 mm) and weight (40 g) make it easy to mount on a wrist band adapted to children. Sounds are synthesized inside the microcontroller that runs an embedded program. Generated sounds can be a combination of continuous tones, intermittent beeps, or playback sounds. Motion sensors provide movement data which are used for triggering or modulating the sounds.

### The rehabilitation training

To test the effectiveness of ABBI, we performed a long rehabilitation protocol of 3 months with children with visual impairment that consisted in several spatial games that the child performed individually with the psychomotor therapist. Several games, described below, were developed with psychomotor therapist and consisted in exercises meant to train his ability to listen and localize sounds in space. Depending on the game to be performed, the child or the psychomotor therapist were asked to wear the bracelet and behave according with the game rules. Some exercises were played in turn by the psychomotor therapist first and by the child immediately after. A variety of sounds were played during the training and these were selected depending on user's preference. Sounds could be pure tones (intermittent or continuous) or preselected playback sounds chosen by the child and stored in the device (e.g., elephant, mosquito, drums). The idea behind sounds customization was to keep the user motivated. Below is reported a detailed description of the games performed in the rehabilitation setting.

- Front-Back Localization: The child is requested to reach the final position of a moving sound that is produced by the psychomotor therapist wearing the ABBI device on the wrist and performing a straight path. The psychomotor therapist and the child are positioned at the same starting point, but the child is oriented toward (front localization) or opposite (back localization) to the psychomotor therapist according to the task requirements. The main objective is to create a detailed auditory map of the space through the requested sensory-motor experience.
- Intercept the sound: the child is requested to intercept a sonorous ball that is thrown by the psychomotor therapist from right to left and vice versa respect to the child position in the room. The child is positioned in the middle of the room and the psychomotor therapist is positioned a bit further on the right or left side in order to throw the ball and allows the child to intercept it with the hands. The main objective is to localize a moving sound source in the space.
- Follow the sound: The child is requested to follow the sound produced by the psychomotor therapist wearing the ABBI device on the wrist and moving randomly in the room with pauses. The psychomotor therapist and the child are positioned at the same starting point. The main objective is to refine the ability to notice and react to directional changes of a moving sound source in space, especially for complex auditory paths (e.g., spirals).
- Grab the sound: the child is requested to run in order to follow and intercept the sound produced by the psychomotor therapist wearing the ABBI device on the wrist and running randomly in the room without pauses. The psychomotor therapist and the child are positioned at the same starting point. The main objective is to promote locomotion and improve the fluency of movements in space.
- Object on the table: The child is requested to find an object positioned on the table in front of him, after it has been moved by the psychomotor therapist wearing ABBI on the wrist. The main objective is to understand the trajectory of the auditory motion in order to reach the goal.
- The lift: the child is requested to localize the sound produced by ABBI in the vertical plane. The psychomotor therapist is in front of the child: he moves the arm on which ABBI is mounted up and down and stops at a specific position asking the child to reach that position. The main objective is to promote the ability to localize sounds in the vertical plane.
- Localization in depth: the child is requested to indicate which of two sounds is closer to his own body. The psychomotor therapist wears ABBI and moves across the room on an imaginary line in front of the child. He turns on the sound on two specific positions of the imaginary line, one close and one far away from the child position. The difficulty of the task is increased across the sessions by making the two sounds closer to each other so that it seems that they come from the same position.
- Reproduction of shapes: The child is requested to reproduce geometrical shapes in space after listening to the sound produced by the psychomotor therapist wearing ABBI and drawing the same shapes while moving in the room. The main objective is to understand how mental representation of geometrical shapes is represented in the real environment through the sound. The same exercise is carried out with the psychomotor therapist drawing shapes on a paper and with the child reproducing it with the pen on a different paper.
- Pampano: The child is requested to jump into one of six rings put on the floor near to each other. The psychomotor therapist wears ABBI on the wrist and jumps into one of the rings. The child needs to understand from which ring the sound came from and jump in the correct ring. Rings are positioned on the floor in a 2 × 3 combination. The main objective is to promote the ability to discriminate among similar sound source positions.
- The path: The child is requested to wear ABBI and walk in the room to position an object in a specific pre-selected position. After the child has positioned the object in the preferred place, the psychomotor therapist removes ABBI from the child's wrist and guides him to the starting position. The main objective is to memorize the path created at the beginning in order to reach and find the object position.
- Mind the obstacles: The child is requested to listen the sonorous path created by the psychomotor therapist who walks in the room wearing ABBI on the wrist and avoid some soft obstacles positioned in the room before the beginning of the exercise. The main objective is to memorize the directional changes made by the experimenter while avoiding obstacles.

### Participants

Fourteen sighted participants (mean age: 3.6, 10 males) and seven visually impaired participants (*n* = 5 low vision children, mean age: 4.4, 4 males; *n* = 2 blind children, mean age: 3.5, 2 males) have been enrolled in the study. Sighted participants reported no visual impairment and a visual acuity better than 9/10. None of the sighted and visually impaired participants had additional sensory disabilities, including hearing disabilities tested with classical audiometer tests during the periodic neuroophthalmological assessment. Table 1 reports the clinical details of the visually impaired children participating in the study: visual acuity values are represented in LogMAR. The main exams used for the functional assessment of visual abilities are light sources method and Early Treatment Diabetic Retinopathy Study with Lea Hyvarinen symbols chart (Lea Symbols® 15-Line Translucent ETDRS-Style Distance Chart). The distance from the chart was 3 m and assessment was performed with both eyes open using a backlit screen. The visual deficit of visually impaired participants has been interpreted according to the International Statistical Classification of Diseases and Related Health Problems (ICD)—10th revision. The term “visual impairment” in category H54 of the ICD classification, comprises category 0 for mild or no visual impairment, category 1 for moderate visual impairment, category 2 for severe visual impairment, categories 3, 4, and 5 for blindness, and category 9 for unqualified visual impairment. The term “low vision” is used for visual acuity less than 0.5–1.3 LogMAR in the better eye with best correction and includes categories 1 and 2. The term “blindness” is used for complete (no light perception) or nearly complete (visual acuity less than 1.3 LogMAR to light perception) vision loss. The participants in our study are defined as “low vision” and “blind” according to these definitions, except two children classified as “low vision” who have a visual acuity of 1.7 LogMAR. In each case the visual deficit was of peripheral origin. The cognitive level of all visually impaired children was assessed with “The Reynell-Zinkin Scales: Developmental Scales for Young Visually Handicapped Children” in two evaluation sessions by two independent therapists. The scales measure mental development of visually impaired children below 5 years old in six areas (social adaptation, sensorimotor understanding, exploration of the environment, response to sound and verbal comprehension, expressive language structure, expressive language content) and provide an age equivalent level of function on each subscale standardized for blind, partially sighted, and sighted children. Psychomotor therapists calculated the mean mental age by merging the evaluation scores for both evaluation sessions and considering the three most intellectually loaded subscales—sensorimotor understanding, response to sound and verbal comprehension, and expressive language content. Among all the children tested, therapists selected the ones whose mental age was considered appropriate for testing, according to the mean scores calculated and the cut-offs proposed by the authors.

**Table 1:** Clinical details of visually impaired children.

**Participant**	**Gender**	**Age (months)**	**Mental age (months)**	**Visual status**	**Visual acuity (LogMAR)**	**Main diagnosis**	**Age at diagnosis**
S1	M	31	27	Early blind	Light perception	Retinopathy of prematurity (V)	Birth
S2	M	48	35	Early blind	NPL^*^	Bilateral anophtalmia	Birth
S3	M	60	57	Low vision	1.22	Bilateral coloboma	1 months
S4	M	64	56	Low vision	1.1	Leber hereditary optic neuropathy (LHON)	5 months
S5	M	54	54	Low vision	1.7	Osteopetrosis	Birth
S6	F	54	51	Low vision	1.22	Microphtalmus and coloboma (sx), anophtalmia (dx)	Birth
S7	F	44	55	Low vision	1.7	Stargradt disease	4 months

*The table shows the clinical details of visually impaired participants: mental age, visual status, visual acuity, main diagnosis, and age at diagnosis. Mental age has been calculated as mean developmental age across all areas covered by the Reynell-Zinkin Scale as explained in the main text. Residual vision has been measured with different methods (light source method, ETDRS Optotype and ERG, ERP, PEV) and is expressed in LogMAR*.

* *NPL (no perception of light)*.

## Results

**Figure 3:**
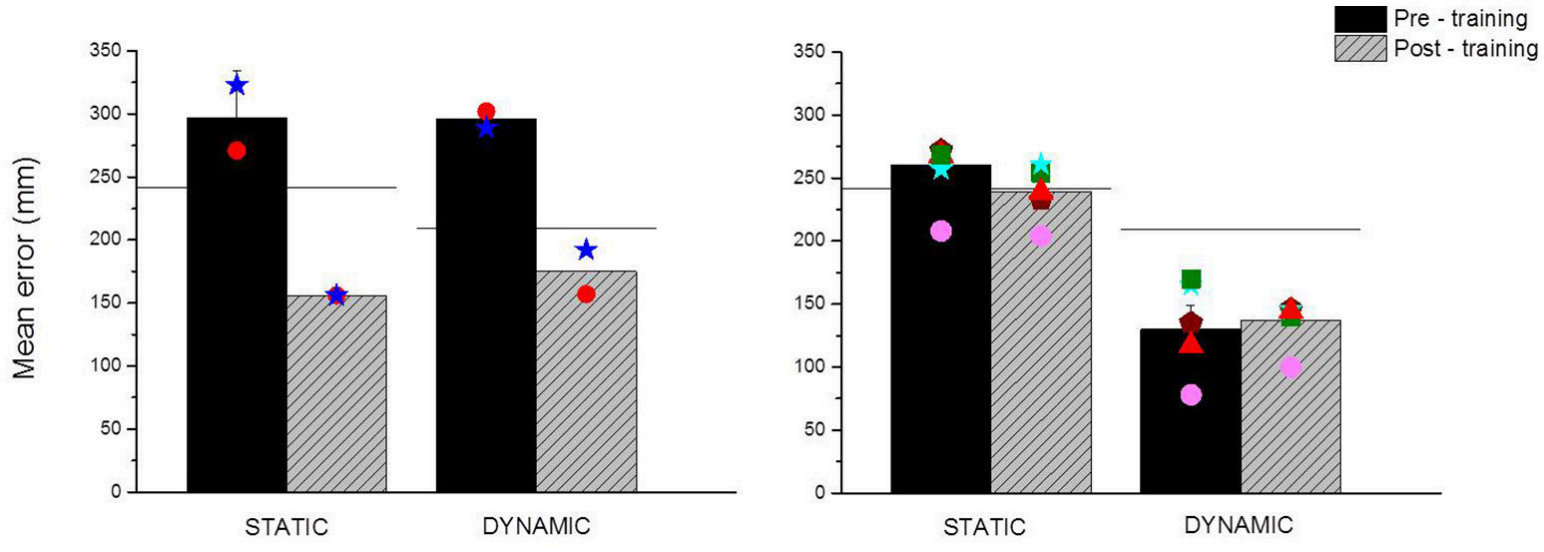
Main outcomes of the rehabilitation training performed with ABBI. The panels show the mean localization error in millimeters obtained by early blind **(Left)** and low vision children **(Right)** in the pre-training (black bars) and post-training (gray bars) sessions of the dynamic and the static tasks. Mean localization error is calculated as the distance between the correct and the perceived position of the sound source. Horizontal black lines represent the spatial performance of age-matched sighted children in the static and dynamic conditions of the localization task. Colored symbols represent the individual data of visually impaired children.

The average results relative to spatial accuracy before and after the training performed with ABBI are presented in Figure 3 for early blind children (left panel) and low vision children (right panel). As a measure of spatial accuracy, we plotted the mean localization error calculated as the average length of the vector that connected the correct and the perceived position of the loudspeaker (mm) for all trials and for each group. The group of early blind children shows a significant improvement in spatial accuracy both in the static (Z = −2.8, *p* < 0.01) and the dynamic (Z = −2.4, *p* < 0.01) conditions of the task indicated by a reduction of the localization error. This finding suggests that the strengthening of audio-motor association provided by the ABBI device is helpful for visually impaired children to refine the spatial representation of the environment. Moreover, as previously demonstrated in Cappagli et al. (2017), early blind children perform overall worse than sighted children in both conditions (Mann-Whitney *U* = 1,161, Z = −2.03, *p* < 0.05 for the static localization task, Mann-Whitney *U* = 1,204, Z = −2.4, *p* < 0.05 for the dynamic localization task), suggesting that the spatial deficit is pervasive and not task specific. Interestingly, after the rehabilitation training early blind children perform even better than sighted children in the static condition of the task (Mann-Whitney *U* = 906, Z = −3, *p* < 0.01), even if this might be also due to the negative influence of the blindfolding procedure on sighted children' accuracy in the pre-training session of the task.

On the contrary, as previously demonstrated in Cappagli et al. (2017), low vision children perform equal to sighted children in the static condition of the task (Mann-Whitney *U* = 6,879, Z = −0.92, *p* = 0.4) and outperformed sighted children in the dynamic condition of the task (Mann-Whitney *U* = 4,460, Z = −5.2, *p* < 0.01), implying that early visual degradation doesn't negatively impact on the ability to localize fixed and moving auditory sound sources. Nonetheless, blindfolding participants might have caused sighted children to perform more incorrectly than in normal situations, and thus similar to low vision children. For this reason, we decided to perform the rehabilitation training also with low vision children even if this group didn't manifest spatial impairments compared to sighted children. Indeed we found that the group of low vision children didn't improve in spatial localization of dynamic (Z = −0.46, *p* = 0.6) and static stimuli (Z = −2.01, *p* = 0.05) after the training. This finding suggests that visual experience, even when degraded from birth as in the case of low vision children, is fundamental for the development of auditory spatial perception. Interestingly, low vision children tend to perform slightly better than controls in the static localization task after the training, even if the difference between the pre- and post-session is not significant. This might suggest that low vision children can benefit from early intervention trainings to compensate for the lack of vision and possibly can develop heightened auditory spatial abilities.

**Figure 4:**
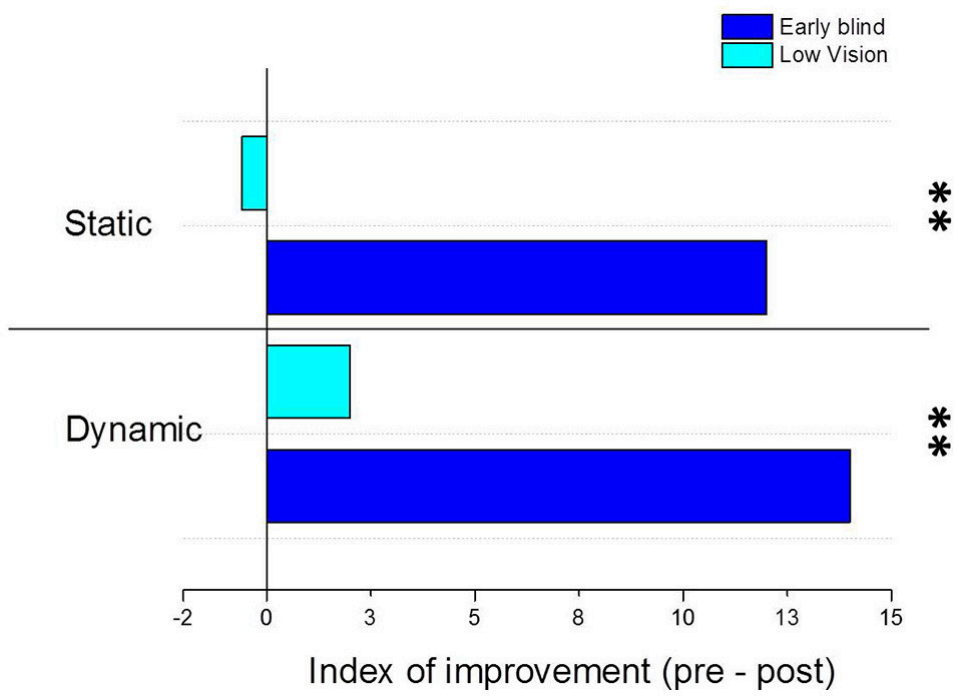
Index of improvement after the training performed with the Audio Bracelet for Blind Interactions (ABBI). The graph shows the index of improvement (in cm) of early blind (dark blue) and low vision (light blue) children in the static and dynamic conditions of the localization task. The index of improvement has been calculated for each group as the difference in terms of mean localization error between the pre-training and the post-training sessions. Positive numbers indicate better spatial performance after the training, negative numbers indicate worse spatial performance after the training. ^**^Indicates *p*-values < 0.01.

The positive effects of the training are further confirmed by calculating the index of improvement across the testing phases (pre-training and post-training) as shown in Figure 4. The graph shows the index of improvement (in cm) of early blind (dark blue) and low vision (light blue) children in the static and dynamic conditions of the localization task, where positive numbers indicate better spatial performance after the training. By comparing the pre-training and the post-training session of early blind and low vision children, we found that a general improvement of the spatial performance is visible for the group of early blind children both for the static [*t*_(85)_ = 4, *p* < 0.01] and the dynamic conditions of the task [*t*_(85)_ = 3.17, *p* < 0.01]. In order to better investigate the spatial improvement after the rehabilitation training, we correlate the index of improvement with visual acuity at the date of testing and with time from vision loss expressed as the sum of months of visual deprivation. We found that only visual acuity correlates with the improvement index both in the static [*F*_(1, 6)_ = 16.5, *R*^2^ = 0.78, *p* < 0.05] and the dynamic [*F*_(1, 6)_ = 7.11, *R*^2^ = 0.6, *p* < 0.05] conditions of the task, confirming that spatial impairments in blindness strongly depend on visual calibration during the development (Gori et al., 2016). On the contrary, the improvement doesn't seem to depend on visual deprivation emergence [*F*_(1, 6)_ = 3.1, *R*^2^ = 0.38, *p* = 0.14 for the static condition; *F*_(1, 6)_ = 5.4, *R*^2^ = 0.5, *p* > 0.05 for the dynamic condition], even when time from vision loss is calculated taking into consideration the age of participants as the sum of months of visual deprivation/sum of months of life [*F*_(1, 6)_ = 0.4, *R*^2^ = 0.08, *p* > 0.5 for the static condition; *F*_(1, 6)_ = 0.76, *R*^2^ = 0.13, *p* > 0.5 for the dynamic condition]. Nonetheless this result might be due to the fact that the age range of participants is very narrow (from 3 to 5 years old) and the time from vision loss parameter doesn't simultaneously take into consideration visual deprivation emergence and current residual vision of participants.

Even if a bigger sample of visually impaired participants would be necessary to draw more general conclusions, these results can have important implications for real-life learning applications. Indeed we provided evidence that learning and plasticity in early life can be highly multisensory and that multisensory stimulation can provide the child with the redundancy he relies on to extract information from the surrounding world. Moreover, the results regarding low vision children further confirm our hypothesis that early visual experience is necessary to develop good audio localization abilities, since overall the group of low vision children didn't show any improvement in spatial performance.

## Discussion

The acquisition of good spatial hearing abilities is the basis for the development of locomotor and social skills in visually impaired children. Nonetheless, recent findings suggest that the development of spatial perception might be prevented in visually impaired children due to the impoverishment of multimodal experiences in their early life. For this reason, an early rehabilitation intervention for visually impaired children should introduce strategies to provide an audio-motor association that compensates for the absence of this visuo-motor association typically required for the development of spatial and social capabilities. The aim of this study was to evaluate the effectiveness of a new rehabilitation device (ABBI) based on the implicit link between action and perception on spatial perception abilities of young visually impaired children. We assessed spatial performance of visually impaired children with an auditory localization task particularly difficult for visually impaired children (Cappagli et al., 2017) and tested possible improvements of spatial perception after the rehabilitation with ABBI. Our results show that the audio-motor training provided by ABBI is helpful in improving spatial hearing in young congenitally blind but not in congenitally low vision children. The finding supports the idea that early and complete loss of one sensory input negatively impacts on the development of perceptual abilities fostered by the missing sensory input (Vercillo et al., 2016) and that the cross-sensory calibration can be restored by using alternative sensory signals (such as audition) when the principal one (e.g., vision for space) is missing.

The last few decades have seen a dramatic rise of interest in the development of assistive technology for visually impaired people, however few products have been commercialized and many prototypes are still not accepted by adult users, mainly because they require intensive training and use complex feedback circuits that might overload sensory, attentional, and memory systems (Gori et al., 2016). The term “assistive technology” is generally used to define devices that satisfy users' needs in terms of assistance: since user safety is always a key issue, assistive devices should not only augment user capabilities, but also ensure their safety and well-being (Csapó et al., 2015). Thus, one of the most important and challenging feature of such technologies is to be appropriate for the sensorimotor capabilities of blind users, both in terms of providing input and interpreting output feedback. Nonetheless the level of complexity of technological devices often represents an obstacle for young children since high attentional resources need to be employed in order to make the devices accessible and usable. Indeed since spatial representations are built during the early years of sensorimotor development and cortical plasticity is maximal during the first years of life (Bremner et al., 2008; Vasilyeva and Lourenco, 2012), an early therapeutic intervention would be fundamental in order to provide effective tools for rehabilitation. Moreover, sensory substitution devices aim by nature at replacing the visual modality and not at rehabilitating the substitute senses, since intact senses are required to process visual properties of the stimuli preventing natural compensation mechanisms. Instead rehabilitation technologies usually enhance functional capacities of residual senses by eliminating or minimizing functional limitations imposed by the disability without explicitly substituting the missing sense. Indeed, devices meant for rehabilitation increase plasticity, leading to structural and functional changes in the brain that foster the reorganization of cortical maps (Johnston, 2009). For this reason, rehabilitation devices are generally adopted during a critical learning period and then removed in order to allow adaptability of the user in the real life after its use. On the contrary, assistive devices are generally adopted during the whole life, reducing the expression of compensation mechanisms spontaneously occurring in the case of sensory loss. In this sense, it would be helpful to develop rehabilitation technologies based on the needs expressed by users and supported by neuroscientific studies evaluating the brain mechanisms that subtend the deficient modality in order to create effective solutions tailored to the problem (Gori et al., 2016). Starting from the limits of the existing technology discussed above, we proposed a new technological device based on the implicit link between action and perception: the ABBI is an audio bracelet that produces a sound when a movement occurs, thus it allows the substitution of the visuo-motor association with an audio-motor association. The idea behind ABBI is that spatial deficits in visually impaired children can be recovered with a multisensory training based on the implicit link between audition and proprioception. For instance, while sighted children can observe their body while performing an action in space, blind children don't have any sensory feedback of their body movements. ABBI can produce an audio feedback that provides spatial sensory feed-back similar to that used by sighted children. The innovation of this system mainly consists in its potential to naturally foster multisensory integration by allowing visually impaired children to experience correspondences between auditory and motor feedback. This device is intuitive, because it does not require extensive learning trainings and it can be introduced early in rehabilitation protocols, thus it is more adaptable compared to existing sensory-substitution devices. Moreover, by exploiting the natural ability to process sensory signals, this system is not intrusive as it would enhance perceptual functions without overwhelming individual sensory and cognitive resources.

In this study, the effects of an extensive but entertaining training with ABBI on the development of spatial hearing have been assessed in a group of seven 3–5 years old visually impaired children. Our results indicate that congenitally blind children improved their spatial performance after the training, while low vision children did not benefit from the use of ABBI. This result can be explained by considering that different sensory feedback are available to totally vs. partially blind children in the first years of life. For instance, low vision children can still benefit from the co-occurrence of visual and motor signals, even if the visual signal is partially compromised, as well as from the association of auditory and motor events. On the contrary congenital blindness that leads to complete loss of visual input totally prevents the possibility to experience multisensory visuo-motor stimuli, leading to a general impoverishment of perceptual experiences. Indeed, as previously demonstrated, while congenital total blindness strongly compromises the ability to localize static and dynamic sound sources, a congenital but only partially degraded visual input can lead to compensatory mechanisms that allow the individual to correctly perceive the position of sounds in space (Cappagli et al., 2017). This finding confirm that vision calibrates other senses to process spatial information when present at birth, while other senses are not trained to encode spatial properties of the world when vision is absent from birth (Vercillo et al., 2016).

The improvement observed in children with congenital and total blindness might be due to a fast refinement of spatial representations as well as to a general boost of attentional resources following the physical and cognitive training with ABBI. Indeed it is well-know that both aerobic and cognitive exercises can improve children's specific executive functions such as working memory and attention (Diamond and Lee, 2011), especially in the case of disability (Stevens et al., 2008). Therefore, the spatial activities which the rehabilitation protocol with ABBI is based on might progressively increase physical and cognitive demands and consequently promote spatial development.

Our results also indicate that low vision children encode the position of dynamic stimuli even better than sighted children. This might be due to the fact that the dynamic condition of the task allows the child to build a more precise auditory representation of the layout, since participants has to wait until the end of the sound motion before responding to the auditory stimulus. Unfortunately there is a lack of studies assessing the ability of blind children to hear sound motion, therefore more focused studies are needed to support this hypothesis.

Moreover these results indicate that the early use of new science-driven devices like ABBI can provide consistent advancements in therapeutic interventions, drastically improving the quality of life of children with visual disabilities. The multisensory training proposed with the ABBI system could foster the ability of individuals to integrate and consolidate auditory and motor signals, leading to an improvement of perceptual abilities. This is probably due to the fact that multisensory protocols are more effective than training protocols based on unisensory stimulus regimes mainly because they facilitate sensory processing due to preexisting congruencies of information coming from the different senses (Shams and Seitz, 2008). Indeed since our behavior is generally guided by the combination of information coming from sensory modalities, unisensory-training protocols used for skill acquisition can be perceived as unnatural settings. Nonetheless, research about the effects of multisensory training on perceptual performance of blind children is lacking, as much of the effort is put in the development of technological devices to replace the role of vision instead of in the improvement of rehabilitation strategies to enhance the potential of intact sensory modalities. A novel approach which aims at developing technological devices to support the improvement of rehabilitation programs would overall fulfill both aspects. The results show that a training based on the association of perceptual and motor functions can facilitate the recalibration of auditory space in blind individuals. The positive effects on spatial perception can be explained by considering that such audio-motor training provides the way to shift from an egocentric to an allocentric reference frame, due to the fact that the sonorous bracelet can be worn both by the blind child and the therapist. Moreover, the training with ABBI increased the opportunities for experiences with independent locomotion and exploration, which might have caused a further refinement of spatial abilities. In agreement with this idea it has been shown that the beginning of self-locomotion facilitates the emergence of allocentric coding in typically developing infants (Cleareld, 2004; Ricken et al., 2004). Finally, a previous study assessing the effects of an audio-motor feedback on spatial localization accuracy of blind adults further confirms this hypothesis, by showing that auditory localization improves even after a very short (2-min) sensorimotor raining (Finocchietti et al., 2017).

Summarizing, we provided evidence that early interventions with the use of science-driven devices can provide consistent advancements in therapeutic interventions. On a broader perspective, the acquisition of sensorimotor capabilities through multisensory trainings might promote benefits not only at perceptual level but also at psychological and cognitive levels. Indeed it has been proposed that cross-correlation of multimodal experiences provides the basis for the development of novel elemental behavioral capacities that are critical for the development of higher-level perceptual and cognitive functions (Thelen and Smith, 1996). Despite the difficulty in recruiting children with total and partial visual impairment at such young age, a bigger sample size would be necessary to draw more general conclusions regarding the positive effects of technology on development. Nonetheless, the results emerging from this study sheds light onto the importance of early and multisensory therapeutic interventions in case of early and pervasive visual loss.

## Conclusion

Our results suggest that a new technological device (ABBI) that associates body motor signals and audio feedback can be used to recalibrate the sense of space in case of vision loss. This approach can be adopted to develop rehabilitation trainings addressed to visually impaired children since the first years of life, leading to a general improvement of their spatial and mobility skills and in turn their ability to interact with the environment and possibly their social inclusion.

## Ethics statement

The study was approved by the ethics committee of the local health service (ASL3 3 Genovese) and parental or adult informed written consent for the study was obtained in all cases before testing the children.

## Author contributions

GC collected, analyzed, and interpreted the data and wrote the article. EC helped recruiting and motivating visually impaired children. SF helped programming the experiments. GB developed the ABBI device and supervised the technological aspects of the study. MG coordinated the study and the development of the ABBI device.

### Conflict of interest statement

The authors declare that the research was conducted in the absence of any commercial or financial relationships that could be construed as a potential conflict of interest.
